# Plant Plastid Engineering

**DOI:** 10.2174/138920210793175912

**Published:** 2010-11

**Authors:** Shabir H. Wani, Nadia Haider, Hitesh Kumar, N.B. Singh

**Affiliations:** 1 Biotechnology Laboratory, Central Institute of Temperate Horticulture, Rangreth, Srinagar, (J&K), 190 007, India; 2 Department of Molecular Biology and Biotechnology, AECS, Damascus P. O. Box 6091, Syria; 3 Department of Plant Breeding and Genetics, Punjab Agricultural University, Ludhiana 141 004, India; 4 Department of Plant Breeding and Genetics, COA, Central Agricultural University, Imphal, Manipur, 795 004, India

**Keywords:** Genetic engineering, genome, plastid transformation, plastome sequencing.

## Abstract

Genetic material in plants is distributed into nucleus, plastids and mitochondria. Plastid has a central role of carrying out photosynthesis in plant cells. Plastid transformation is becoming more popular and an alternative to nuclear gene transformation because of various advantages like high protein levels, the feasibility of expressing multiple proteins from polycistronic mRNAs, and gene containment through the lack of pollen transmission. Recently, much progress in plastid engineering has been made. In addition to model plant tobacco, many transplastomic crop plants have been generated which possess higher resistance to biotic and abiotic stresses and molecular pharming. In this mini review, we will discuss the features of the plastid DNA and advantages of plastid transformation. We will also present some examples of transplastomic plants developed so far through plastid engineering, and the various applications of plastid transformation.

## INTRODUCTION

Genetic material in plants is distributed into nucleus and the chloroplast and mitochondria in the cytoplasm. Each of these three compartments carries its own genome and expresses heritable traits [1, 2]. The chloroplast is one of organelles known as plastids in plant cells and eukaryotic algae [3]. According to Verhounig *et al*. [4], plastids and mitochondria are derived from formerly free-living bacteria and have largely prokaryotic gene expression machinery. The plastid (biosynthetic centre of the plant cell) carries out photosynthesis, in plant cells and eukaryotic algae, which provides the primary source of the world's food [3]. There are other important activities that occur in plastids. These include sequestration of carbon, production of starch, evolution of oxygen, synthesis of amino acids, fatty acids, and pigments, and key aspects of sulfur and nitrogen metabolism [5]. In spite of the prokaryotic past of the plastids, their gene expression has very different regulatory mechanisms from those operating in bacteria [6]. There are up to 300 plastids [7] in one plant cell. The plastid genome (plastome or plastid DNA, ptDNA), 1,000–10,000 copies per cell [8], contrasts strikingly with the nuclear DNA. In most species, plastids are usually strictly maternally inherited [9] in most (80%) angiosperm plant species [10, 11]. It is also not influenced by polyploidy, gene duplication and recombination that are widespread features of the nuclear genomes of plants [12, 13]. Therefore, ptDNA varies little among angiosperms in terms of size, structure and gene content [14]. Currently 170 chloroplast genomes from different species have been completely sequenced (NCBI Organelle Genome Resources; http://www.ncbi.nlm.nih.gov/genomes/). These also include many agriculturally important plant species like rice [15, 16] maize [17] sugarcane [18], wheat [19, 20], tomato [21], and mungbean [22] (Table **1**). The plastid genome was determined to be circular double-stranded DNA through construction of complete genome maps. Its size ranges from 120.000 to 180.000 base pairs, depending on the species, that encode ~120 genes. Each plastid genome constitutes in almost all higher plant species of a large single copy (LSC), a small single copy (SSC), and duplication of a large (~25 kb) region (IRs) in an inverted orientation [2, 23].

Genetic engineering has been experienced mostly in the nuclear genome [24, 25]. Inserting transgene(s) into the nuclear genome, however, has led to an increasing public concern of the possibility of escape of the transgene through pollen to wild or weedy relatives of the transgenic crops [26]. Scientists argued that since plastids are compared with prokaryotes, they can take up DNA as in bacterial transformation using naked DNA (http://www.studentsguide.in/ plant-biotechnology-genomics/transplastomic-plants-chloroplast-engineering/advantages-of-chloroplast-transformation. html). Therefore, during the past few years, researchers have begun to evaluate application of plastid engineering (transformation) in plant biotechnology as a viable alternative to conventional technologies for transformation of the nuclear DNA [27]. Recently, plastids have become attractive targets for genetic engineering efforts [28]. Plants with transformed plastid genomes are termed transplastomic [29].

**Fig. (1):**
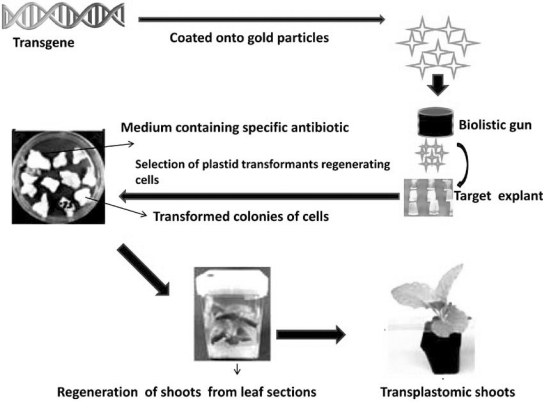
Plant plastid engineering.

Diekmann *et al*. [30] believe that in order to develop plastid engineering, obtaining plastid genome sequences is crucial, and that efficient sequencing requires pure plastid DNA. The authors, therefore, developed a simple and inexpensive method to obtain plastid DNA from grass species by modifying and extending protocols optimized for the use in eudicots. Plastid engineering involves the targeting of foreign genes to the plastid's double-stranded circular DNA genome instead of chromosomal DNA [31], and as a consequence the production of the foreign protein of interest (Fig. **1**). The advancement in the particle gun mediated transformation has enabled targeting the plastid genome for developing transgenic plants against many biotic (e.g. insects and pathogens) and abiotic stresses (e.g. drought and salinity), which reduce the plant productivity. Improving the quality of fruits has been another main target in plastid transformation [32]. 

Plastid transformation was first achieved in a unicellular alga, *Chlamydomonas reindhartii* [33]. In 1990, Sváb *et al*. [34] reported the first successful chloroplast transformation of a higher plant (tobacco). This was followed by transformation of the plastid genome in tobacco by many researchers [35, 36]. Recently, tobacco plastid has been engineered to express the E7 HPV type 16 protein, which is an attractive candidate for anticancer vaccine development [37]. Similarly, a protocol for plastid transformation of an elite rapeseed cultivar (*Brassica napus* L.) has been developed [38]. More recently, a method for plastid transformation in eggplant (*Solanum melongena* L.) has been reported with pPRV111A plastid expression vector carrying the *aadA* gene encoding aminoglycoside 300-adenylyltransferase [26]. The authors believe that this may open up exciting possibilities to introduce and express novel genes in the engineered plants *via *plastid transformation for agronomic or pharmaceutical traits. Up to date, plastid transformation has been extended to many other higher plants, such as *Arabidopsis thaliana *[39], potato [40, 41], tomato [1, 42], *Lesquerella fendleri*, a kind of oilseed *Brassicaceae* [43], oilseed rape, [38, 44], petunia [45], lettuce [46], soybean [47], cotton [48], carrot [49], rice [50], poplar [51], tobacco, [28, 52, 53], mulberry , [54] and eggplant [26] (see review written by Wang *et al*. [3]).

Two interesting applications of plastid transformation were carried out by (i) [55] for the construction of a tobacco master line to improve Rubisco engineering in plastids, and (ii), [4] who explored the possibility of engineering riboswitches (natural RNA sensors that regulate gene expression in response to ligand binding) to function as translational regulators of gene and transgene expression in plastids.

The dominant trait that attracted the most attention for plastid transformation has been herbicide tolerance [28, 35, 56, 57]. Roudsari *et al*. [28] revealed the production of high level glyphosate tolerant plants (*N*. *tabacum*) through biolistic transformation of plastids by introduction of a mutated herbicide-tolerant gene coding for EPSP synthase. Plastid transformation is routine, however, only in tobacco and the efficiency of transformation is much higher in tobacco than in other plants [58]. Lee *et al*. [50] discussed the major obstacles to the extension of plastid transformation technology to other crop plants which includes regeneration *via *somatic embryogenesis. 

## ADVANTAGES OF PLASTID ENGINEERING

Production of transgenic plants, at laboratory level or commercially, has traditionally been mainly through expression of transgenes in the nucleus [24, 25]. Among the ecological concerns raised about genetically engineered organisms is that transgenes could move ("transgene flow", the process of transgene movement by recurrent hybridisation) *via *pollen from the crop and into relatives growing in natural or semi-natural communities [59]. Such concerns have led to a new field of transgene containment [60, 61]. 

Since plastids are inherited maternally in the majority of angiosperm species, they would therefore not be found in pollen grains of corps. Insertion of transgenes, therefore, into the plastid genome has the potential of preventing gene flow *via *pollen. Hence, genes expressed in the plastome will not be transferred through pollination to weedy or wild relatives of the transgenic crop. Bansal and Sharma, [62] believes that there is little risk of any transgene flow *via *pollen from transplastomic plants to the neighboring weedy or wild relatives since plastids are almost always maternally transferred to the next progeny. The authors suggested that plastid transformation could be a method of choice for generating improved transgenics in crops that grow along with their weedy or wild relatives in the same geographical region, such as rice, sorghum, cucurbits, solanaceous crops, *Vigna *and *Cajanus *species, and various *Brassica *crops*.* Therefore, the focus of many researchers has shifted to plastid engineering [26], rather than nuclear transformation. Singh *et al*. 2010 [26] reported that engineering of the plastid genome is gaining momentum as an attractive alternative to nuclear transformation.

Ruf *et al.* [60] believe that plastid transformation is considered as a superb tool for ensuring transgene containment and improving the biosafety of transgenic plants. However, they pointed out that plastid transformation would only be effective as a biocontainment measure when applied on a landscape scale if it were combined additional mechanisms such as mitigating genes genetic use restriction technology, and/or male sterility [63]. In a recent study, it has been demonstrated that the use of plastid transformation would provide an imperfect biocontainment for GM oilseed rape (*Brassica napus* L.) in the United Kingdom [64]. In another study, Allainguillaume *et al*. [65] revealed that chloroplast transformation may slow transgene recruitment in two settings, but actually accelerate transgene spread in a third. Plastid transformation has become an attractive alternative to nuclear gene transformation due to several other advantages [3]. The high ploidy number of the plastid genome allows high levels (up to 1-40% of total protein) of protein expression or expression of the transgene [28]. Daniell *et al*. [66] and Hou *et al*. [44] reported that while nuclear transgenes typically result in 0.5 - 3% of total proteins, concentration of proteins expressed by plastid transgenes is much higher; up to 18%. The greater production of the expressed protein is possible because plastid transgenes are present as multiple copies per plant cell, and they are little affected by phenomena like pre- or post-transcriptional silencing. Other advantages of plastid engineering are the capacity to express multiple genes from polycistronic messenger RNA (mRNA) [31], and the absence of epigenetic effects and gene silencing [40]. 

Wang *et al*. [3] believe that transgene stacking in operons and a lack of epigenetic interference allowing stable transgene expression. Added to that, plastid transformation is more environmental friendly than transformation of the nuclear DNA for plant engineering because it eliminates the possibility of toxic transgenic pollen to nontarget insects [67]. Adverse effects of toxic proteins might be minimized by plastid compartmentalization but in case of nuclear transformation, toxic proteins accumulating within the cytosol might result in serious pleiotropic effects. Further, the expression of the transgene in case of plastid transformation is more uniform compared to that of trangenes inserted into the nuclear genome. Although there is a major drawback in the engineering of plastid gene expression, which is the lack of tissue-specific developmentally regulated control mechanisms [3], the many advantages of plastid engineering stated above attracted researchers to engineer the plastid genome to confer several useful agronomic traits, and hence the number of species whose plastome can be transformed continues to expand [68].

## GENE DELIVERY INTO PLASTIDS

Gene delivery into the plastome was initially done by *Agrobacterium* mediated method [Block *et al.*1985] [69]. The discovery of biolistic DNA delivery led to plastid transformation *via *particle gun [Sanford 1990] [70]. In this method, the *Escherichia coli* plasmids contain a marker gene and the gene of interest is introduced into plastids. The foreign genes are inserted into plasmid DNA by homologous recombination *via *the flanking sequences at the insertion site [66]. Polyethylene glycol (PEG) mediated transformation of plastids was also utilized [71, 72]. PEG-mediated transformation of plastids requires enzymatically removing the cell wall to obtain protoplasts, then exposing the protoplasts to purified DNA in the presence of PEG. The protoplasts first shrink in the presence of PEG, then lyse due to disintegration of the cell membrane. Removing PEG before the membrane is irreversibly damaged reverses the process. Biolistic delivery is the routine system for most laboratories, as manipulation of leaves, cotyledons, or cultured cells in tissue culture is a simple practice than the alternative PEG treatment of protoplasts [58]. Recently, particle gun mediated plastid transformation has been demonstrated in rapeseed using cotyledons as explants [38].

The major difficulty in engineering plastid genome for production of transplastomic plants is in generating homoplasmic plants in which all the plastids are uniformly transformed, for that takes a long process of selection, thus hampering the production of genetically stable transplastomic plants (e.g. rice). This is due to the presence of about 10-100 plastids, each of which has up to 100 copies of the plastid genome, in one cell, that does not allow achieving homoplastomic state [73]. It was also stated that getting high level of protein expression, even though the gene copy number is high, is another problem. In 2005, however, Nguyen *et al*. [41] described the generation of homoplasmic plastid transformants of a commercial cultivar of potato (*Solanum tuberosum* L.) using two tobacco specific plastid transformation vectors, pZS197 (P*rrn*/*aadA*/*psbA3*′) and pMSK18 (*trc*/*gfp*/P*rrn*/*aadA*/*psbA3*′). Similarly, Liu *et al.* [74] were able to develop homoplasmic fertile plants of *Brassica oleracea* L. var. capitata L. (cabbage). Among other higher plants of which fertile homoplasmic plants with genetically modified plastid genomes have be been produced are *Nicotiana tabacum* (tobacco), *Nicotiana plumbaginifolia* (texmex tobacco), *Solanum lycopersicum* (tomato), *Glycine max* (soybean), *Lesquerella fendleri* (bladderpod), *Gossypium hirsutum* (cotton), *Petunia hybrida* (petunia), and *Lactuca sativa* (lettuce) [68]. The amino glycoside 3-adenylyltransferase *(aad*A) gene, which confers dual resistance to spectinomycin-streptomycin antibiotics, is still the selectable marker that is routinely used efficiently for plastid transformation [58, 75, 76]. Since the antibiotic resistant genes used in transformation are not desirable in the final products, different strategies have been developed to eliminate the necessity of using such selectable markers [56, 77]. 

## IMPROVEMENT OF SOME AGRONOMIC TRAITS BY PLASTID ENGINEERING

Apel & Bock, [42] demonstrated the potential of plastids genome engineering for the nutritional enhancement of food crops when they enhanced carotenoid biosynthesis in transplastomic tomatoes by induced lycopene-to-provitamin A conversion. The transplastomic technology could also be useful for engineering agronomic traits including phytoremediation [78] reversible male sterility [79], and tolerance/resistance of stresses such as diseases, drought, insect pests, salinity and freezing that can severely limit plant growth and development [3]. 

Since plastids are transferred mostly through the “maternal inheritance” as identical copies, and hence a female plant transfers identical copies to all the seeds it produces without changes from one generation to the next, an important promise for applying plastid transformation for industry is the stable passing on to the next generation of the foreign DNA [80]. Therefore, the plastid genome has also been utilized for metabolic pathway engineering and in the field of molecular farming (the production of drugs and chemicals through engineered crops) [27, 68] for the expression and production of biomaterials and biopharmaceuticals in plants, human therapeutic proteins, and vaccines for use in humans or animals (reviewed in [26, 27, 50]. Singh *et al*. [26] believe that for such applications, plastid transformation technology offers solutions to the ecological and technical problems associated with conventional transgenic technologies such as outcrossing and transgene silencing.

### Insect Resistance

Transformation of the nuclear genome of plants with genes (e.g. *Bt* genes) to confer insect resistance gives very low levels of expression unless extensive modifications are carried. Whereas, introduction of the same genes into the plastid genome results in high levels of toxin accumulation as the plastid genome is bacterial in origin [81]. Therefore, the insect resistance genes were investigated for high-level expression from the plastid genome [3]. Hence, when insect resistance genes are expressed into the plastid genome, leaves of these transplastomic plants proved highly toxic to herbivorous insect larvae. 

One of the major advantages of introducing the *Bt* toxin into the plastid genome is the high levels of toxin accumulation (3% – 5% of total leaf protein as compared to > 0.2% of total soluble protein through nuclear genome transformation) [82]. Achievement of stable transformation of the plastid genome and transforming plastids in species other than tobacco (*Nicotiana tabacum*) are some of the hurdles for widespread adaptation of this technique [81]. Despite of overwhelming odds, many attempts have been made to produce transplastomic plants expressing *Bt* toxin for increased resistance against insect pests. [83] generated soybean plastid transformants expressing *Bacillus thuringiensis Cry1Ab* protoxin. Similarly, Chakrabarti *et al*. [84] reported the control of potato tuber moth (*Phthorimaea operculella*) by incorporating a truncated *Bacillus thuringiensis* *cry9Aa2* gene in the plastid genome. The authors observed high-level expression (about 10% of total soluble protein) of the *cry *gene from the plastid genome which resulted in severe growth retardation. 

Over-expression of the *cry2Aa2 *operon in plastids was proved effective in allowing a broad-spectrum of protection against a range of pests [81]. In cabbage, the *cry1Ab* gene was also successfully transferred into the plastid genome [85]. Expression of *cry1Ab* protein was detected in the range of 4.8–11.1% of total soluble protein in transgenic mature leaves of the two species. Insecticidal effects on *Plutella* *xylostella* were also demonstrated in *cry1Ab* transformed cabbage. In an attempt to increase insect resistance in transgenic rice plants, a synthetic truncated *cry1Ac* gene was linked to the rice *rbcS* promoter and its transit peptide sequence (tp) for plastid-targeted expression [86]. Use of the *rbcS-tp* sequence increased the *cry1Ac* transcript and protein levels by 25- and 100-fold, respectively, with the accumulated protein in plastids comprising up to 2% of the total soluble proteins. The high level of *cry1Ac* expression resulted in high levels of plant resistance to three common rice pests, rice leaf folder, rice green caterpillar, and rice skipper, as evidenced by insect feeding assays. It was concluded that targeting of *cry1Ac* protein to the plastid using the *rbcS:tp* system confers a high level of plant protection to insects. Several other* cry* proteins have also been expressed in plastids of tobacco [83, 84, 87, 88] and rice [50] (see Table **2**). 

### Disease Resistance

Plastid engineering offers a new and effective option in development of plant varieties which are resistant to various bacterial and fungal diseases. In tobacco, introduction of *MSI-99* gene, an antimicrobial peptide, into plastids resulted in transplastomic plants resistant to fungal pathogen *Colletotrichum destructive* [89]. The plastids expressed *MSI-99* at high levels and showed 88% (T1) and 96% (T2) inhibition of growth against *Pseudomonas syringe*, which is a major plant pathogen. In another study,* Agrobacterium* mediated transformation was used to develop tobacco plants carrying *argK* gene, which encodes ROCT [90]. Since OCT in plant cells is produced in the plastid, *argK* was fused to the plastid transit sequence of the pea rubisco small subunit (*rbcS*) gene for localized expression of the enzyme. The ROCT enzyme produced by the transgenic tobacco showed greater resistance (83-100%) to phaseolotoxin compared to the wild-type OCT (0-22%). When phaseolotoxin was applied exogenously to the leaves of plants, chlorosis was observed in 100% of wild-type tobacco, but not seen in the leaves of the transgenic tobacco plants carrying the *argK* gene from *P. syringae* pv*. phaseolicola*. Transgenic tobacco plants that constitutively expressed both *entC* and *pmsB* in the plastid have also been reported [91] where transformation was accomplished through biolistic methods. The transgenic tobacco plants expressing these bacterial genes showed accumulation of salicylic acid that were up to 1000 times higher than that observed in wild-type tobacco. When challenged with the fungus *Oidium lycopersicon*, the transgenic tobacco plants showed increased levels of resistance compared to the wild-type plants. It was revealed that the transgenic plants generated did not show any adverse effects due to the high level expression of salicylic acid. Thus gene transfer in plastids can provide a significant protection from various bacterial and fungal diseases. 

### Drought and Salinity Tolerance

Transgenes that confer tolerance to abiotic stress may permanently transfer from transgenic crops to the nuclear genome of their weedy relatives which may result in drought tolerant superweeds [92] when the gene is inserted into the nuclear genome and the transgenic plant outcross with relative weeds. There is a great potential, therefore, for the genetic manipulation of key enzymes involved in stress metabolism in plants within plastids. Because plastid genomes of major crops including cotton and soybean have been successfully transformed, this offers an exciting new approach to create transgenic plants with abiotic stress tolerance [93]. Therefore, the authors believe that there appears to be tremendous potential for increasing tolerance in plants to a number of stresses by expression of appropriate genes within plastids due to the maternal inheritance of transgenes that confer tolerance to abiotic stress. Plastid engineering had been successfully applied for the development of plants with tolerance to salt, drought [94] and low temperature [reviewed in 3]. Djilianov *et al*. [95] demonstrated that enhanced tolerance to abiotic stresses has been achieved when the gene was directed to plastid genome.

Among many strategies used for development of abiotic stress tolerance in plants, the over-expression of compatible osmolytes like glycinebetaine was found to be successful [24]. Initial attempts for producing transplastomic plants through the introduction of CMO (Choline monooxygenase) and betaine aldehyde dehydrogenase (BADH) pathway were made in tobacco. Tobacco plants were transformed with cDNA for BADH from spinach (*Spinacia oleracea*) and sugar beet (*Beta vulgaris*) under the control of CaMV 35 S promoter. The BADH was produced in plastids of tobacco. Betaine aldehyde was converted to betaine by BADH, thus conferring resistance to betaine aldehyde. In another attempt, cDNA for choline monooxygenase from *Spinacia oleracea* was introduced into tobacco and the enzyme thus synthesized was transported to its functional place i.e., plastids. But the leaves of tobacco accumulated betaine at a very low concentration i.e., 10-100 folds lower [96]. The reason for insufficient synthesis of betaine most probably was the absence of engineered BADH activity in plastids. Therefore, both CMO and BADH need to be present in the plastids for efficient synthesis of betaine in transgenic plants which do not accumulate glycinebetaine. In carrot (*Daucus* *carota*), homoplasmic transgenic plants exhibiting high levels of salt tolerance were regenerated from bombarded cell cultures *via *somatic embryogenesis [48]. BADH enzyme activity was enhanced 8-fold in transgenic carrot cell cultures, grew 7-fold more, and accumulated 50- to 54-fold more betaine than untransformed cells grown in liquid medium containing 100 mM NaCl. Transgenic carrot plants expressing BADH grew in the presence of high concentrations of NaCl (up to 400 mM), the highest level of salt tolerance reported so far among genetically modified crop plants. Further, a gene for CMO, cloned from spinach (*Spinacia oleracea*) was introduced into rice through *Agrobacterium* mediated transformation. The level of glycinebetaine in rice was low to the expectations. The author has given several reasons for the low productivity of rice and low glycinebetaine accumulation. Firstly, the position of spinach CMO and endogenous BADH might be different and secondly the catalytic activity of spinach CMO in rice plants might be lower than it was in spinach [97]. Transplastomic plants constitutively expressing *BvCMO* under the control of the ribosomal RNA operon promoter and a synthetic T7 gene G10 leader were able to accumulate glycinebetaine in leaves, roots and seeds, and exhibited improved tolerance to toxic level of choline and to salt/drought stress when compared to wild type plants. Transplastomic plants showed higher net photosynthetic rate and apparent quantum yield of photosynthesis in the presence of 150 mM NaCl [98]. Thus, it can be concluded that glycinebetaine has a role as compatible solute and its engineering into non-accumulations will be a success only if both CMO and BADH pathways are introduced and if the localization of both CMO and BADH is in plastids. Very recently, George *et al.* [99] demonstrated how a chloroplast-localized and auxin-induced glutathione S-transferase from phreatophyte *Prosopis juliflora* conferred drought tolerance on tobacco. For more examples of conferring tolerance to abiotic stress to plants *via *plastid engineering, see review by [3].

## PRODUCTION OF BIOPHARMACEUTICALS AND VACCINES IN PLANTS

Mulesky *et al*. [100] pointed out two reasons that make using crops to produce drugs interesting for industry. These are (i) crops can be employed more efficiently in this process than animals or bacteria, with a larger output achieved with fewer resources, and (ii) the oral delivery of the drugs produced to people and animals is easier. A third reason is the high-level production of antigens for use as vaccines and their tests for immunological efficacy in animal studies [3]. The hyper-expression of vaccine antigens or therapeutic proteins in transgenic chloroplasts (leaves) or chromoplasts (fruits/roots) and antibiotic-free selection systems available in plastid transformation systems made possible the oral delivery of vaccine antigens against cholera, tetanus, anthrax, plague, and canine parvovirus, [101-104] and reviews of [102, 105, 3 and 106] explained why plastid engineering can be regarded as an attractive strategy and environmentally friendly approach for the production of vaccines, therapeutic proteins, and biomaterials, and provided some examples. Kumar & Daniell [107] described various techniques for creating plastid transgenic plants and their biochemical and molecular characterization. They also provided suitable examples for application of chloroplast genetic engineering in human medicine.

Wang *et al*. [3] also discussed applying plastid transformation for metabolic pathway engineering in plants, the production of biopharmaceuticals, and marker gene excision system and how plastid transformation can be applied to study RNA editing. Similarly, Hefferon [67], considers plastid engineering as a valuable tool that gives enormous promise for the production of biopharmaceuticals and vaccines, because higher level of the protein expressed by the transgene inserted into the plastid genome can be achieved. Many vaccine antigens, which played a key role for the prevention of infectious diseases, and biopharmaceutical proteins, have been expressed at high levels *via *the chloroplast genome [108] and they proved to be functional using *in vitro* assays in cell cultures*. *[109] stated that production of therapeutic proteins in plastids eliminates the expensive fermentation technology, and that the oral delivery of plastid-derived therapeutic proteins eliminates cold storage, cold transportation, expensive purification steps, and delivery *via *sterile needles, and hence decrease their cost. The main goal for applications of plastid transformation by the biotech industry is molecular pharming, and food production is considered as only a secondary target [80]. 

To create an edible vaccine, selected desired genes should be introduced into plants and then inducing these altered plants to manufacture the encoded proteins. Like conventional subunit vaccines, edible vaccines are composed of antigenic proteins and are devoid of pathogenic genes. Plastids of green plants as bioreactors for the production of vaccines and biopharmaceuticals are of great potential as indicated from a number of published studies [110-114]. The significance of using plants as production platforms for pharmaceuticals is due to the low production and delivery costs, easy scale-up and high safety standards regarding less risk of product contamination with human pathogen [27]. Keeping in view the high efficiency of plastids to express foreign genes, it is meaningful to explore this property of plastids for the production of proteinaceous pharmaceuticals, such as antigens, antibodies and antimicrobials. The candidate subunit vaccine against *Clostridium* *tetani*, causing tetanus was the first plastid-produced antigen that proved to be immunologically active in experimental animals [111]. In this initial attempt, fragment C of the tetanus toxin (TetC), a non-toxic protein fragment, was expressed from the tobacco plastid genome which resulted in high levels of antigen protein expression (30% of the plant’s total soluble protein (TSP)). Anthrax is an acute infectious disease caused by the spore-forming bacterium *Bacillus* *anthracis*. Significant development has been achieved towards the production of plastid-based vaccine for this infectious disease. Expression (14% of the plants TSP) of the *pagA* gene encoding the protective antigen (PA) from the tobacco plastid genome gave rise to stable antigen protein [112, 113]. The plastid-derived PA was equally effective in cytotoxicity assays as the bacterial protein produced in *B. anthracis*. The potential of plastid transformation as an alternative tool to produce high levels of HIV-1 Nef and p24 antigens in plant cells have been also demonstrated [115]. Different constructs were designed to express the p27 Nef protein either alone or as p24-Nef or Nef-p24 fusion proteins. All constructs were utilized to transform tobacco (cv. Petite Havana) plastids and the transplastomic lines. Analysis of p24-Nef and Nefp24 fusion proteins showed that both can be expressed to relatively high levels in plastids. As the best results in terms of protein expression levels were obtained with the p24-Nef fusion protein, the correspondent gene was cloned in a new expression vector. This construct was introduced into the tobacco and tomato plastid genomes. Transplastomic tobacco and tomato plants were analyzed and protein accumulation was found to be close to 40% of the leaf’s total protein. Transcript and protein accumulation were analyzed in different ripening stage of tomato fruit and green tomatoes accumulated the fusion protein to 2.5% of the TSP [115]. Recently, a strategy for plastid production of antibiotics against pneumonia* Streptococcus pneumonia *has been outlined [116]. The authors describe it as a new technique for high level expression (to up to 30% of the plant’s TSP) of antmicrobial proteins that are toxic to *E. coli*. It was also shown that the plastid-produced antibiotics efficiently kill pathogenic strains of *Streptococcus pneumoniae*, the causative agent of pneumonia, thus providing a promising strategy for the production of next-generation antibiotics in plants. In 2007, Chebolu and Daniell [117] achieved a stable expression of Gal/GalNAc lectin of *Entamoeba histolytica *in transgenic chloroplasts and immunogenicity in mice towards vaccine development for amoebiasis. Shao *et al*. [118] reported the expression of the structural protein E2 of classical swine fever virus (CSFV), which has been shown to carry critical epitopes CFSV E2 gene in tobacco chloroplasts. In another study on tobacco, plastid transformation of the high-biomass tobacco variety `Maryland Mammoth` has been assessed by McCabe *et al*. [119] as a production platform for the human immunodeficiency virus type 1 (HIV-1) p24 antigen. Similarly, Meyers *et al*. 2008 [120] revealed the usefulness of plastid signal peptides in enhancing the production of recombinant proteins meant for use as vaccines. Transgenic plastids were also proved efficient for high-yield production of the vaccinia virus envelope protein A27L in plant cellsdagger by Rigano *et al*. [121], who revealed that chloroplasts are an attractive production vehicle for the expression of OPV subunit vaccines. Very recently, Youm *et al*. [122] were able to produce the human beta-site APP cleaving enzyme (BACE) *via *transformation of tobacco plastids. The authors argued that the successful production of plastid-based BACE protein has the potential for developing a plant-based vaccine against Alzheimer disease. Because recombinant extra domain A from fibronectin (EDA) could be used as an adjuvant for vaccine development, [104] aimed to express EDA from the tobacco plastome as a promising strategy in molecular farming. Tobacco plastids transformation was also evaluated by Lentz *et al*. [123] for the production of a highly immunogenic epitope containing amino acid residues 135-160 of the structural protein VP1 of the foot and mouth disease virus (FMDV). The authors concluded that this technology allows the production of large quantities of immunogenic proteins.

In spite of this huge success in applying plastid engineering for molecular pharming, Ho & Cummins [73] referred to several risks of plastid engineering in producing GM pharmaceuticals that are associated with its advantages. For more details on the use of plastid engineering for biotechnology applications, see review by Verma & Daniell [5]. Other valuable reviews are available on (a) generation of plants with transgenic plastids, summary of our current understanding of the transformation process and highlights on selected applications of transplastomic technologies in basic and applied research [124], (b) Progress in expressing proteins that are biomedically relevant, in engineering metabolic pathways, and in manipulating photosynthesis and agronomic traits and the problems of implementing the technology in crops [125], (c) plastid transformation in higher plants [56], (d) the characteristics, applications of chloroplast genetic engineering and its promising prospects, [126], (e) Engineering the chloroplast genome [127] (f) exciting developments in this field and offers directions for further research and development, [128] (g) the expression of resistance traits, the production of biopharmaceuticals and metabolic pathway engineering in plants [27], (h) chloroplasts as bioreactors, and whether we can replace plants with plants [129], (i) how plastid transformation played an important role in understanding the RNA editing [3] and (j) comparison of opportunities and challenges between nuclear and plastid genetic engineering of plants [130]. 

## CONCLUSIONS

Up to date, many transgenes have been successfully introduced into the plastid genome of model plant tobacco and many other important crop plants for various agronomic traits. Initially, this technology was limited to model plant species, but now it has been extended to some other important crops. Still there are many agronomically important cereals crops in which plastid engineering has not yet been standardized. Plastid transformation has been proved to result in high levels of transgene expression. It has also provided a baseline for production of proteinaceous pharmaceuticals, such as antigens, antibodies and antimicrobials in a cost effective manner. Bock [27] believes that a great progress has been achieved over years in investigating the mechanisms that govern transgene expression from the plastid genome and in using this technology for biotechnological applications. We agree with the author that "the routine use of plastid engineering in biotechnology is still a long way off, but would surely benefit the humanity in the near future". There is no doubt that plastid engineering holds a great potential in plant biotechnology; but like every new technology, there are some challenges which need to be addressed before its widespread adoption. Among other important factors to be solved are the protein purification and expression level control.

## Figures and Tables

**Table 1:** A List of Sequenced Plastomes of Some Agriculturally Important Plants

Plant species	Base pairs	Reference
*Arabidopsis thaliana*	154,478	[131]
*Brassica napus*	-	[132]
*Citrus sinensis*	155,189	[133]
*Coffea arabica*	-	[134]
*Cucumis sativus*	155,293	[135]
*Daucus carota*	155,911	[136]
*Ficus sp.*	-	[137]
*Glycine max*	152,218	[138]
*Gossypium barbadense*	160,317	[139]
*Gossypium hirsutum*	160,301	[140]
*Helianthus annuus*	151,104	[141]
*Hordeum vulgare, Sorghum bicolor*	-	[142]
*Lycopersicon esculentum*	155,460	[21]
*Manihot esculenta*	-	[143]
*Morus indica*	156,599	[144]
*Musa acuminata*	-	[145]
*Nicotiana tabacum*	155,943	[146]
*Oryza sativa*	134,551	[15]
*Oryza nivara*	134,494	[16]
*Phaseolus vulgaris*	150,285	[147]
*Saccharum officinarum*	141,182	[18]
*Solanum tuberosu*	155,298	[148]
*Spinacia oleracea*	150,725	[149]
*Triticum aestivum*	134,545	[19, 20]
*Vitis vinifera*	160,928	[150]
*Zea mays*	22,784	[17]
*Jatropha curcas*	163,856	[151]
*Vigna radiata*	151,271	[22]

**Table 2:** A List of Some Transplastomic Plants that were Engineered for Various Agronomic Traits

Plant species	Gene introduced	Reference
*Nicotiana tabacum*	*rrn16*	[152]
*Nicotiana tabacum*	*nptII*	[153]
*Nicotiana tabacum*	*uidA*	[154]
*Nicotiana tabacum*	Human somatotropin *(hST)*	[155]
*Nicotiana tabacum*	*cry*	[88]
*Nicotiana tabacum*	*cry9Aa2*	[84]
*Nicotiana tabacum*	*Bar & aadA*	[156]
*Nicotiana tabacum*	Cor 15a-FAD7	[157]
*Nicotiana tabacum*	*rbcL*	[55]
*Nicotiana tabacum*	*DXR*	[158]
*Nicotiana tabacum*	*aadA & gfp*	[60]
*Nicotiana tabacum*	Delta(9) desaturase	[159]
*Nicotiana tabacum*	*AsA2*	[160]
*Nicotiana tabacum*	*PhaG & PhaC*	[161]
*Nicotiana tabacum*	*gfp*	[4]
*Nicotiana tabacum*	*A1AT*	[162]
*Arabidopsis thaliana*	*aadA*	[39]
*Solanum tuberosum*	*aadA & gfp*	[40]
*Oryza sativa*	*aadA & gfp*	[50]
*Solanum lycopersicon*	*aadA*	[1]
*Solanum lycopersicon*	*Lyc*	[42]
*Brassica napus*	*aadA & cry1Aa10*	[44]
*Brassica napus*	*aadA*	[38]
*Lesquerella fendleri*	*aadA & gfp*	[43]
*Daucus carota*	*dehydrogenase (badh)*	[48]
*Gossypium hirsutum*	*aphA-6*	[49]
*Glycine max*	*aadA*	[47]
*Petunia hybrida*	*aadA & gusA*	[45]
*Lactuca sativa*	*gfp*	[46]
*Brassica olerace*a	*gus & aadA*	[163]
Lettuce	*gfp*	[164]
*Populus alba*	*gfp*	[51]
*Brassica oleracea*	*aadA & uidA*	[74]
*Beta vulgaris*	*aadA & uidA*	[165]
*Crocus sativus*	*CstLcyB1& CstLcyB2a*	[166]
*Solanum melongena*	*aadA*	[26]
*Arabidopsis thaliana*	*pre-Tic40-His*	[167]
*Zea mays*	*ManA*	[168]
